# Expedient in Difficult Labor

**Published:** 1875-03

**Authors:** 


A NOVEL EXPEDIENT IN A DIFFICULT LABOR.—To illustrate what a quick-witted man may do in an emergency, Dr. Cotting gave the account of a sea-captain who was crossing the Atlantic with a load of immigrants, one of whom was a woman in labor. At the end of the first day he consulted his medical library without getting any satisfactory information. At the end of the second day the woman was getting exhausted, the pains were diminishing, the scalp protruding, "everything blocked up," general condition of things deplorable. Appealed to in this emergency, the captain placed the woman on her side, flexed the exposed thigh forcibly upon her abdomen, placed his knee against the lower end of the sacrum, at the same telling her to strain away for once and all, which being done, the child was immediately born. *Boston Med. and Surg. Jour.*, Jan. 28, 1875, p. 103.

